# The influence of insight on risky decision making and nucleus accumbens activation

**DOI:** 10.1038/s41598-023-44293-2

**Published:** 2023-10-11

**Authors:** Maxi Becker, Yuhua Yu, Roberto Cabeza

**Affiliations:** 1 Department of Psychology, Humboldt University Berlin, 10099 Berlin, Germany; 2 Department of Psychology, Northwestern University, Chicago, IL 60637 USA; 3 Center for Cognitive Neuroscience, Duke University, Durham, NC 27708 USA

**Keywords:** Human behaviour, Cognitive neuroscience, Emotion, Motivation, Reward

## Abstract

During insightful problem solving, the solution appears unexpectedly and is accompanied by the feeling of an AHA!. Research suggests that this affective component of insight can have consequences beyond the solution itself by motivating future behavior, such as risky (high reward and high uncertainty) decision making. Here, we investigate the behavioral and neural support for the motivational role of AHA in decision making involving monetary choices. The positive affect of the AHA! experience has been linked to internal reward. Reward in turn has been linked to dopaminergic signal transmission in the Nucleus Accumbens (NAcc) and risky decision making. Therefore, we hypothesized that insight activates reward-related brain areas, modulating risky decision making. We tested this hypothesis in two studies. First, in a pre-registered online study (Study 1), we demonstrated the behavioral effect of insight-related increase in risky decision making using a visual Mooney identification paradigm. Participants were more likely to choose the riskier monetary payout when they had previously solved the Mooney image with high compared to low accompanied AHA!. Second, in an fMRI study (Study 2), we measured the effects of insight on NAcc activity using a similar Mooney identification paradigm to the one of Study 1. Greater NAcc activity was found when participants solved the Mooney image with high vs low AHA!. Taken together, our results link insight to enhanced NAcc activity and a preference for high but uncertain rewards, suggesting that insight enhances reward-related brain areas possibly via dopaminergic signal transmission, promoting risky decision making.

## Introduction

Sometimes during problem solving, the solution appears as a surprise and is accompanied by an AHA! experience. This sudden comprehension of a non-obvious solution often requiring a novel problem representation is considered an *insight*—a form of creative cognition^1,2^. People do not always have an AHA! when they come up with ideas or solve problems. But when they do, the idea or solution finding feels internally rewarding including a feeling of suddenness and certainty^3–5^. The affective component of the AHA! experience is functionally important because it motivates future behaviour related and unrelated to the content of the insight^6–10^. Recent evidence suggests that people bias towards risky options when they make a monetary choice after solving a verbal puzzle with accompanied AHA!.^10^. Risky decision making in this context describes the subject’s preference to choose a high-reward, high uncertainty option over a low-reward, low uncertainty option. In the current study, we investigated the cognitive and neural mechanisms of the motivational role of AHA! in decision making.

Several human and animal studies have linked internal reward and positive emotional arousal to dopaminergic signal transmission in the ventral striatum, specifically the Nucleus Accumbens (NAcc)^11–15^. Dopaminergic activity is an important reward signalling mechanism that marks the significance of information and modulates subsequent behaviour. Phasic dopaminergic bursts in the NAcc play a central role in learning rewarding contingencies^16^ and in risky decision making^17,18^. fMRI studies have shown that NAcc activity precedes and predicts financial risk seeking^19,20^. Further data on rodents and humans, including Parkinson’s disease patients, indicate that dopaminergic drugs increase risk-taking behaviour^21–25^ by modulating the perceived attractiveness of risky options^24,26,27^. Dopamine is thought to signal a reward prediction error (difference between predicted and expected reward) specifically for unexpected rewards. The unexpected reward gates Hebbian plasticity in the striatum, facilitating the repetition of rewarding actions, in turn biasing behaviour towards effortful and risky actions to acquire rewards^28^. Note, previous work has suggested that the AHA! experience is also related to a reward prediction error as it reflects the subjective response to a better than expected outcome, i.e. the sudden solution is unexpected followed by positive arousal and internal reward^29^.

Given the above mentioned evidence, we therefore hypothesize that insight-related increase in high reward/high uncertainty decision making could be due to increased reward-based dopaminergic signalling in the NAcc ultimately modulating choice behaviour. In fact, the relevance of striatal dopamine for creative cognition has been discussed before^30,31^, and there is first evidence for insight-related BOLD activity increase in the NAcc in verbal puzzles^4,32^. However, the insight-related reward signalling and decision-making modulation have not been studied under the same paradigm. To fill this gap and investigate our hypothesis, we conducted two studies.

In the first study, we tested the generalizability of the insight-related risky decision making bias, originally shown by Yu and colleagues^33^. To this end, we conducted a preregistered online study using a different insight-eliciting task than Yu et al.^33^. We asked participants to solve Mooney images—hard-to-recognize, high-contrast photos of real-world objects^6,34,35^, and to rate their AHA! experience after each solution. To assess the influence of the AHA! experience on risky decision making, we adopted a risk elicitation task that used real monetary rewards following each AHA! rating (see^33^). The participants could choose between a smaller fixed (deterministic) payout and a “risky” payout where they have 20% chance of receiving a bigger payout but 80% chance of receiving zero. We predicted that participants would be more likely to choose the higher but riskier payout after solving a Mooney image with, compared to without, accompanied AHA!.

In the second study, we investigated whether insight during the Money identification task would be associated with increased dopaminergic activity in the reward system. For this, we used data from an fMRI study of the Mooney identification paradigm (see^36^). As a proxy for reward-related dopaminergic signal transmission, we measured event-related BOLD activity in NAcc^14,37–39^. We predicted that NAcc activity would be higher for solutions with, compared to without, accompanied AHA!.

## Methods

### Study 1: online experiment

The goal of the first study was to test the generalizability of insight-related risky decision making, previously found during a word association task^10^. We aimed to replicate this effect using an insight paradigm under a pictorial solving task, namely Mooney images. Given we had precise hypotheses based on this prior research, this study was pre-registered (https://aspredicted.org/p7ir2.pdf). We hypothesised that participants will favour a higher monetary reward with uncertainty over a lower fixed reward after solving a Mooney image with a high, compared to a low, AHA! (insight-related risky decision making). Note that the outcome of the monetary reward is unrelated to the visual insight problems itself other than the time proximity.

#### Participants

Relying on the effect size from a study investigating risky decision making in insight^10^, we estimated a sample size of n = 150 (power = 80%, alpha error = 5%). Due to the high sample size, we conducted this study as an online format for an English speaking population (USA: n = 65; Germany: n = 84; others: n = 9). We recruited a final sample size of n = 158 [age (in years): range = 18–45, 93 females: M = 28.7; 63 males: M = 32.6] using the online platform Mechanical Turk and the student online platform PESA of the Humboldt University Berlin. The local ethics committee of the Humboldt University Berlin approved of the study. Informed consent was obtained from all participants and they received monetary compensation according to their achieved bonus pay. Inclusion criteria were no prior neurological or psychiatric diseases, age between 18 and 45 years, English language proficiency. To ensure comprehension of task instructions, participants were required to self-report a minimum proficiency level of "Upper intermediate English," scoring at least 4 on a scale ranging from 0 (indicating no English proficiency) to 6 (indicating English as a mother tongue). Subjects were also excluded from further analyses if they showed no variance in their AHA! rating (e.g. they always rated the same number, n = 1) or in their risk choice (e.g. they always chose the fixed or the high risk option, n = 53), following previous work^10^ and as specified in the preregistration. 54 subjects were excluded from the study based on the last criterion resulting in a final sample of n = 103 [age (in years): range = 20–45, 66 females: M = 28.8; 37 males: M = 32.7; USA: n = 41; Germany: n = 56; others: n = 6, based on self-assessment]. Note, the proportion of excluded subjects based on zero variance in their risk choice is comparable to the proportion reported by Yu and colleagues^33^. We conducted additional analysis with data including participants with zero variance, the results did not change significantly (see Supplementary Material; Table S1). All research was performed in accordance with the relevant guidelines/regulations and in accordance with the Declaration of Helsinki.

#### Materials and procedure

**Figure 1:**
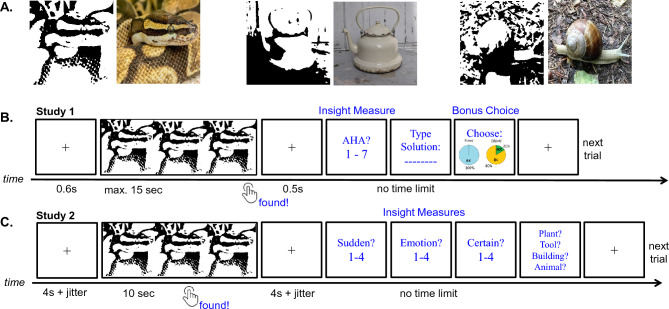
Example Mooney images and Experiment Procedures in Study 1 and 2. (**A**) Examples of Mooney images (left) and their non-abstracted real-world equivalents (from left to right: snake, kettle, snail). (**B**) Experimental design for Study 1. After solving a Mooney image, participants rated their AHA! experience on a single scale (see section “Assessing insight”) and subsequently typed the name of the Mooney object. Then, they were presented with a bonus choice to assess their risk preference immediately after solving. Participants chose between two options: a fixed payout (6¢) and a risk payout with a 20% chance to receive the high amount (e.g. 25¢), by clicking on the graphical representation. (**C**) Experimental design for fMRI Study 2. After solving a Mooney image, participants reported their insight experience with three separate ratings and subsequently chose the category that fits the solution (1 out of 4).

We utilised high-contrast, black and white images (henceforth Mooney), which have been successfully used to induce visual insight before^6,34,35^. Those images depict a concrete object but recognition is difficult due to its high contrast, with solution times ranging from several seconds up to minutes^34^. Visually regrouping the abstracted shapes can lead to sudden object recognition, often involving an insight^6,35^. Examples of abstracted Mooney images used in this study and their real-world equivalents are depicted in Fig. 1A. The stimulus material for Study 1 comprised 80 Mooney images, which was a subset of images used in Study 2 (see Materials & Procedure for Study 2^36^). The 80 Mooney images were chosen based on mean solution time, accuracy, and insight probability to match them (on average) with the fMRI sample from Study 2.

Before participants executed the visual insight task with subsequent bonus choice, their payout amount was first customised in a *risk baseline survey*. It is important to customise the payout amount to have a sensitive measure to small insight-related shifts in preference, despite the wide range of individual differences in risk preference (see Frey et al.^40^).

##### Risk baseline survey

This survey was a multiple price list^41^ adapted from Yu and colleagues^33^. The survey consisted of five levels. At each level, participants were asked to choose either a fixed payout (6¢) or a risk payout: an 80% chance of receiving either nothing (0¢) or a 20% of receiving a high amount that varied across five levels (level 1: 15¢, level 2: 25¢, level 3: 35¢, level 4: 45¢ or level 5: 55¢). Participants generally prefer the fixed payout at level 1. As the amount of the higher payout increases they are expected to switch to the risk payout at a certain level indicating their risk preference. The baseline was defined as the risk payout immediately after the switch. For example, if a subject chose the fixed amount (6¢) over the 20% chance of 35¢, but then preferred the 20% chance of 45¢ over the fixed amount (6¢), the baseline was set to 45 ¢. The survey allows us to infer the payout amount in the bonus such that the participant feels indifferent towards the fixed vs. the risky payout (see below).

If a participant did not switch from the fixed to the risk payout or if they showed inconsistent switching (i.e. switching back to the fixed payout at a higher level)^33^, we could not infer a valid indifference level, and would exclude this participant for further analysis. However, no participants were excluded based on this criterion.

##### Mooney identification paradigm with bonus choice

After the risk baseline survey, participants read the instruction for the Mooney identification task with bonus choice and the explanation of the AHA! experience. They were given two examples to practise the task.

The procedure for the main task looked as follows: After a 600 ms fixation cross, the Mooney image would be presented for max. 15 s. Subjects were instructed to press a solution button immediately after they solved the Mooney image, i.e. identified the object in the Mooney image. If subjects failed to press the solution button, after 15 s a new trial would start. If they pressed the solution button, after 500 ms they were asked to rate to what degree they experienced an AHA! (see section “Assessing insight” below) and subsequently name/type the Mooney object. Finally, they were presented with the bonus choice. Here, participants were asked to choose between a fixed payout (6¢) and a risk payout with a 20% chance to receive the high amount (e.g. 25¢). The high amount was customized for each participant based on the baseline survey, and was fixed throughout the experiment. Subsequently a new trial would start. Note, identical to Yu and colleagues (2022), the bonus choice was presented in picture form to help subjects understand the probability of receiving the monetary reward in the fixed as well as in the risk payout condition (see Fig. 1C). The position of both payout options on the screen (left or right) was randomized for each trial to avoid automated responses. There was no time limit for naming the Mooney object, AHA! rating or the bonus choice.

To discourage participants from reporting incorrect solutions for the sake of receiving bonus, we told participants at the beginning of the experience that only correct solutions qualified for a bonus. However, to their ignorance, every solution (correct and incorrect) was counted as a bonus.

##### Assessing insight

Insight is usually measured via self-ratings of the AHA! experience which was previously quantified in a binary way as present or absent (Jung-Beeman et al.^42^; for review Kounios and Beeman^43^). However, recent studies have shown that the AHA! experience is a continuous phenomenon consisting of multiple components, in particular (1) positive emotional response upon solution finding, (2) perceived suddenness of the solution and (3) certainty about the correctness of solution^3,44^. For this reason, we assessed the AHA! experience on a continuous scale (from 1 to 7) and described the concept to the participants as follows: “Insight describes the *sudden* and *certain* understanding of a problem that often involves an AHA!-experience. The AHA!-experience is the *feeling of pleasure* when the solution comes to you in a *sudden* manner.” Note, the description entails the same three concepts of suddenness, feeling of pleasure and (to a lesser degree) certainty but, in contrast to Study 2, here we asked the subjects for a combined rating. We assessed the combined AHA! experience in this experiment because too many individual ratings may diminish the potential transient effect of insight upon risk decision. We analyzed the insight rating both as a continuous measure and a binarized variable via median split into high (HI-I) and low (LO-I) insight in order to make our results comparable to prior work.

#### Analyses

To investigate the effect of the AHA! experience on subsequent risky decision making, two binomial mixed effect models were estimated with bonus choice (binary, fixed vs. risky) as dependent measure. The baseline model (I) included a variable for accuracy (acc), trial number (trial#) as well as two random intercepts for subject and item to control for random variance (see equations below). Accuracy was included as covariate into the model because high and low insight trials significantly differ in accuracy as has also been consistently found and discussed in previous studies^3,45–48^. The trial number was included to control for potential order effects on risk-based decision making. The full model (II) was identical to the baseline model but additionally included a factor that differentiated between two conditions—solved trials with high (HI-I) as well as low insight (LO-I) as independent measure.

I. Risk choice ~ acc + trial# + (1|ID) + (1|item) + Ɛ.
II. Risk choice ~ acc + trial# + insight (HI-I, LO-I) + (1|ID) + (1|item) + Ɛ.

*Note*: *acc* accuracy, *trial#* trial number.

To test whether risk choice is linearly modulated by the strength of the experienced AHA!, the previous analysis was repeated with insight as continuous (1–7) instead of binary measure (see Table 2). P-values for the nested mixed effects models were calculated via log-likelihood tests. All analyses were conducted in R (v4.2.0) and the random mixed effects models were estimated using the glmmTMB-function (v1.1.3).

### Study 2—fMRI experiment

To probe for a mechanistic explanation of the increased risky decision making after insight, in Study 2 we tested whether insightful problem solving during the Mooney identification paradigm is associated with heightened activity in NAcc, which has been widely used as a proxy for reward-related dopaminergic activity^14,37–39^. We reanalyzed the results of an fMRI dataset using the same Mooney identification paradigm as in Study 1^36^; note, the analyses and results of Study 2 have not been reported). To assure that the results from Study 1 and 2 can be reasonably compared, we additionally performed control analyses testing whether there are significant differences in group characteristics between the two studies.

#### Participants

A total of 38 participants [age (in years): M = 25.3, range = 20–34, 23 females: M = 25.0; 15 males: M = 25.7] was recruited via an online student platform in Berlin and subsequently scanned. Inclusion criteria were between 18 and 35 years, no prior neurological or psychiatric diseases, German as mother language, normal or corrected vision, and MRI compatibility. The local ethics committee of the Humboldt University Berlin approved of the study. Prior to study begin, informed consent was obtained from all participants and they received monetary compensation according to their time on task. Six subjects had to be excluded from further analyses due to technical issues at the scanner (N = 1), due to pathological findings in brain anatomy (N = 2) or too excessive head movement in the scanner (N = 3). This resulted in a final sample of N = 32 [age (in years): M = 25.2, range = 19–33; 20 females: M = 24.7; 12 males: M = 26.2]. All research was performed in accordance with the relevant guidelines/regulations and in accordance with the Declaration of Helsinki.

#### Materials and procedure

The stimulus material consisted of 120 Mooney images and was partially taken from Imamoglu and colleagues^34^ (2013, N = 34). To ensure MRI compatibility, we only included Mooney images with minimal accuracy of 30% (*M* = 56%, *SD* = 18.2%), solution time of min. 2 s and max. 13 s (*M* = 6.23 s, *SD* = 3.11 s) and sufficient variance in self-rated insight experience (*M* = 62%, *SD* = 17%, range [20–100%]). Because not enough of those images from Imamoglu met those inclusion criteria, we additionally manually created and online piloted new Mooney images that met those criteria.

##### Mooney identification paradigm

The participants completed two MRI sessions on two consecutive days and each session comprised two blocks (á 30 trials). The order of the presented images per trial was randomized, and the blocks and sessions were counterbalanced between subjects. In the scanner, the experiment was presented on a black screen (resolution 1280 × 960 pixel) using Matlab (2016a) and Psychtoolbox-3 (v3.0.17^49^. Participants indicated their solution with their right index finger on a four-button response box as soon as they found a solution. The trials of the Mooney identification paradigm were identical to the one in Study 1 with a few differences (see Fig. 1b,c): (1) The Mooney image was presented for a total of 10 s instead of 15 s, and it would stay on the screen for 10 entire seconds due to methodological reasons, not relevant for the current study (for more details, see^50^). (2) The fixation cross before and after the stimulus presentation was jittered (4 s on average). (3) Insight was assessed via three consecutive ratings (no time limit): suddenness (“On a scale from 1 to 4: Did the solution come to you in a sudden or more gradual manner?”), emotion (“On a scale from 1 to 4: How strong was your positive emotional response upon solution?) and certainty (“On a scale from 1 to 4: How certain are you that the solution is correct?”) (see section below). Finally (4), instead of typing the solution, the participants had to choose the correct out of four response categories e.g. “Objects in the House”, “Reptiles or Insects”, “Human Being” or “Body & Health”, to identify the solution (no time limit).

The Mooney images were originally presented together with 30 anagram riddles per trial in an interleaved fashion. However, anagram data was not analyzed in the current study^50^.

##### Assessing insight

Similar to Study 1, insight was assessed via the AHA! experience on a continuous scale (1–4) but here split into its three main components (positive emotion, certainty and suddenness; see previous section: Mooney identification paradigm) in line with previous research^3,44^. For better comparability with Study 1, we summed up all three scales into one continuous compound insight measure ranging from 3 to 12. Similar to Study 1, the AHA! experience is analyzed as a continuous but also as a binary measure via median split into high (*HI-I*) and low (*LO-I*) insight. Furthermore, to test if NAcc activity is linked to the positive emotion as predicted—or other components of AHA! as well, we conducted an exploratory analysis on NAcc activity in relation to the three scales.

#### fMRI data acquisition

Functional and structural images were collected on a Siemens Magnetom Prisma 3 T scanner (Erlangen, Germany) using a standard 64-channel head coil. All sequences were adapted according to the Human Connectome Project^51^. Structural images were obtained using a three-dimensional T1-weighted magnetization prepared gradient-echo sequence (MPRAGE) (TR = 2500 ms; TE = 2.22 ms; TI = 1000 ms, 208 slices, acquisition matrix = 240 × 256 × 167, FoV = 256 mm, flip angle = 8°; 0.8 mm^3^ voxel size). Multiband functional images were collected using a T2*-weighted echo planar imaging (EPI) sequence sensitive to blood oxygen level dependent (BOLD) contrast (TR = 800 ms; TE = 37 ms, 72 slices; voxel size = 2.0 mm^3^; flip angle = 52°; FoV = 208 mm; acquisition matrix = 208 × 208 × 144; multi-band accel. factor = 8). Additionally a spin echo field map was acquired to account for the B0 inhomogeneities (TR = 8000 ms; TE = 66 ms; flip angle = 90°, 72 slices; FoV = 208 mm; 2 mm^3^ voxel size, acquisition matrix = 208 × 208 × 144).

#### fMRI preprocessing

Functional as well as structural images were preprocessed with fMRIPrep version 20.2.5 using default parameters^50,52^. This included skull stripping (OASIS template) of T1-weighted images, segmentation and reconstruction of brain surfaces using recon-all from FreeSurfer v6.0.1^53^. Additionally, functional images were not slice time corrected but distortion corrected using an implementation of the TOPUP technique (Andersson et al. 2003). Importantly, functional and structural images were co-registered but not normalized into standard space but rather preprocessed in native space (T1).

#### Region-of-interest analysis

**Figure 2:**
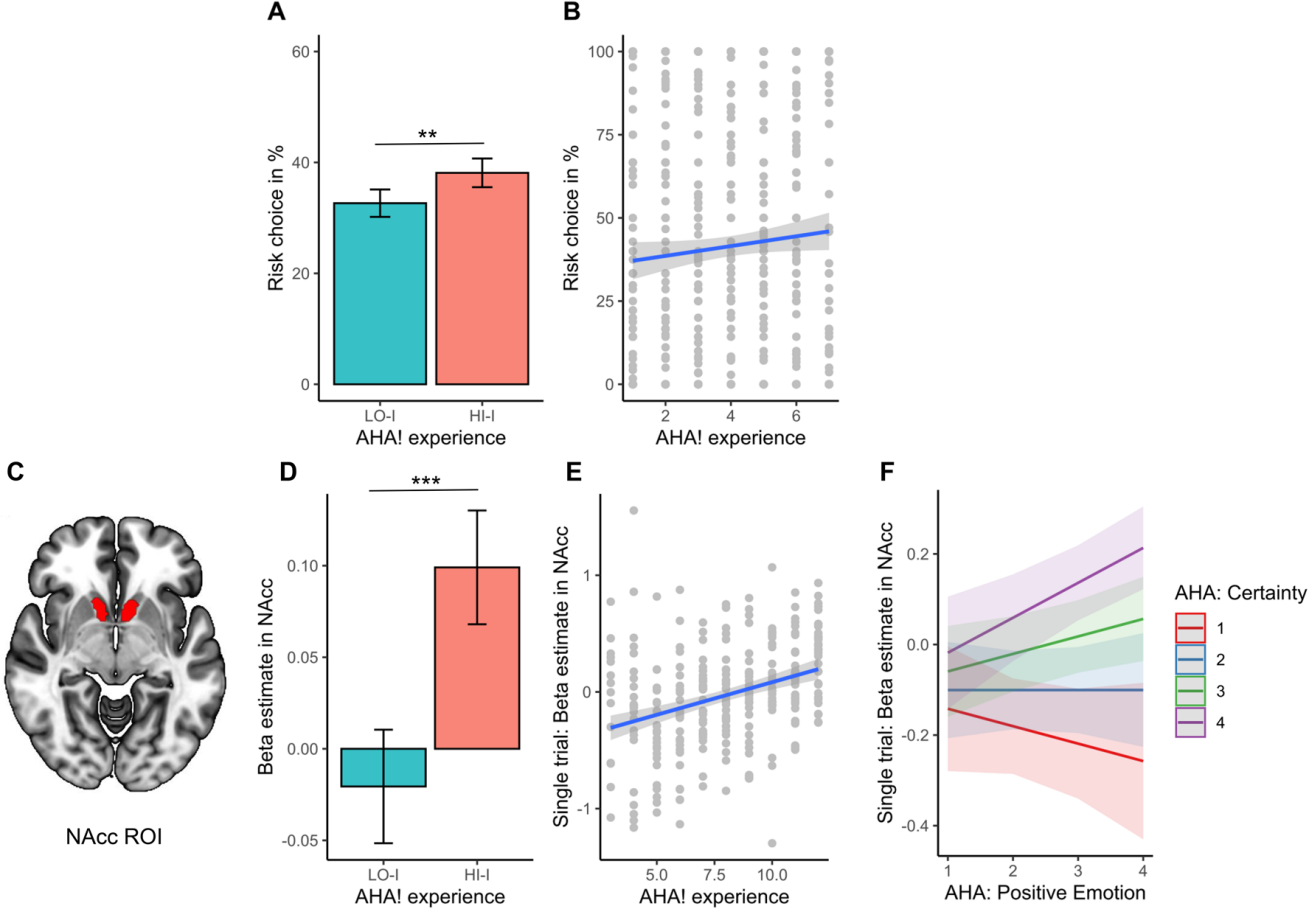
Influence of insight (AHA!) on risky decision making (Study 1) and activity in Nucleus Accumbens (NAcc, Study 2). The values for Panel (**A**) & (**D**) represent estimated marginal means ± SEM. **p < 0.005, ***p < 0.001. The values for Panel (**B**) & (**E**) represent raw values with regression line ± SEM. *NAcc* Nucleus Accumbens. (**A**, **B**) Study 1—Influence of AHA! experience (binary (**A**) and continuous (**B**) measure) on bonus choice. (**D**, **E**) Study 2—Influence of AHA! experience (binary (**D**) and continuous (**E**) measure) on BOLD activity in NAcc. Note, the continuous AHA! experience measure in Study 1 (**B**) consisted of a single scale from 1 to 7 and in Study 2 (**E**) it is a sum measure (1–12) of its three main components (*positive emotion, suddenness, certainty*) each ranging from 1 to 4. (**F**) Interaction between AHA! experience components: *positive emotion* and *certainty* predicting NAcc activity during solution.

We chose a region-of-interest (ROI) approach to estimate insight-related increase in BOLD activity in NAcc (see Fig. 2C). To exclude potential smoothing and normalization artefacts due to the small volume of the NAcc, all analyses were conducted in native space. The left and right NAcc ROI were taken from the FSL Harvard Oxford Atlas^54^ and transformed into native space for every subject using an ANTs transformation command (*antsApplyTransforms*^55^).

To analyze BOLD changes for the contrast high insight (HI-I) > low insight (LO-I), all events per condition were pooled together per subject, session and block (1). *Averaged Beta Analysis*) as implemented in SPM12 using the standard parameter settings (Welcome Department of Cognitive Neurology, London, UK). However, to estimate the influence of the continuous AHA! experience measure on NAcc activity while controlling for accuracy on a trial by trial basis, we additionally calculated a *Single Trial Beta Analysis* (2)^56^; see below).

##### Averaged beta analysis (binary AHA! measure)

To evaluate the effect of insight on NAcc activity, we contrasted brain activity during *HI-I* and *LO-I* trials. The first-level analysis was conducted in the framework of general linear models (GLMs) following a mass univariate approach as implemented in SPM using standard parameter settings. Regressors were created by convolving the onsets of the respective conditions with the canonical hemodynamic response function (HRF) and their first and second temporal derivative. The time series were corrected for baseline drifts by applying a high-pass filter (128 s) and for serial dependency by an AR(1) autocorrelation model.

For the first-level analysis, a total of 13 separate regressors was created. We modelled the solution button presses for the Mooney images in two different conditions: *HI-I*^1^, *LO-I*^2^. To reduce variance from the implicit baseline, we added two nuisance regressors; one for all solution button presses of the anagram trials^3^ (which are not reported here) and another one for all remaining button presses^4^ (related to the insight ratings and selecting a solution category). We assumed that subjects already processed the solution shortly before the button press^42^. Therefore, all events were modelled for one second before until button press. Additionally, we separately modelled six motion parameters [5:10] and separately modelled the mean for each of the four runs [11:13].

We used the *marsbar* toolbox using its default parameter settings to extract the averaged beta values representing the respective onset regressors from the NAcc ROI per session and block^57^. The mask for NAcc was extracted from the FSL Harvard-Oxford (HO) Atlas^54^.

To statistically (second level) compare insight-related mean activity differences in NAcc brain activity, we estimated two nested mixed effect models in R similar to Study 1. In the baseline model (I., see equation below), the beta value for NAcc activity was predicted by the following covariates: gender, accuracy (to maintain the same set of covariates employed in all previous analyses, we included accuracy as covariate but, note, this value is aggregated, representing an average accuracy score per participant per condition, i.e., HI-I or LO-I), gender, run and ROI (left versus right). Note, due to laterality effects in NAcc, we estimated the left and right NAcc separately^58,59^. Run is a factor specifying the order of the respective run order (1:4, 2 blocks, 2 sessions) and was included as covariate to control for potential differences between the sessions and blocks. Additionally, a subject variable served as random intercept. The full model was identical to the baseline model and additionally included an insight factor that differentiated between solved trials with high (HI-I) and low (LO-I) accompanied insight.

I. Beta value ~ acc + run + gender + ROI + (1|ID) + Ɛ.
II. Beta value ~ acc + run + gender + insight(HI-I, LO-I) + ROI + (1|ID) + Ɛ.

*Note*. *acc* accuracy.

Posthoc marginal mean differences between conditions were estimated using the emmeans package (v.1.7.5) and p-values for the nested mixed effects models were calculated via likelihood-ratio tests via the R’s anova function (v4.2.0).

##### Single Trial Beta Analysis (continuous AHA! measure)

To estimate the influence of the continuous AHA! experience measure on NAcc activity on a trial by trial basis, we additionally conducted a single trial analysis in subject space within SPM’s first level analysis pipeline^56^. We chose this analysis because it flexibly allows for statistical control of multiple covariates on a trial level in the same statistical mixed model framework as had already been carried out in Study 1.

During first level analysis, beta values for each solution event were estimated. These beta values were calculated by specifying the solution event of interest for each Mooney image as an onset regressor of 1TR (0.8 s) beginning with the solution button press. We added an additional nuisance regressor for all remaining button presses (related to the insight ratings and selecting a solution category) as a one second event. Additionally, we modelled six motion parameters and the mean for each of the four runs. All events were convolved with a canonical hemodynamic response function (HRF). Finally, a high-pass filter with a 128 s cut-off period was applied to control for baseline drifts.

For statistical (second level) analysis, the resulting beta values for the left and right subject-specific NAcc ROI corresponding to each solved Mooney image were extracted and predicted via a mixed effects model (see equations below). The baseline model (I) consisted of covariates similar to covariates of no interest in Study 1: accuracy, trial number (trial#), gender and ROI (left or right Nacc) including a random subject and item intercept. The full model was identical to the baseline model but additionally included a continuous (sum) measure for the AHA! experience a predictor. P-values for the nested mixed effects models were calculated via likelihood-ratio tests.

I. Beta value ~ acc + trial# + run + ROI + (1|ID) + (1|item) + Ɛ.
II. Beta value ~ acc + trial# + run + ROI + insight(continuous) + (1|ID) + (1|item) + Ɛ.

*Note*. *acc* accuracy, *trial#* trial number, *ID* subject.

##### Exploratory single trial beta analysis with individual insight components

Due to the AHA! sum measure, we cannot rule out the possibility that *suddenness* or *certainty,* instead of *positive emotion,* are driving the observed effects in NAcc. Because we had a specific hypothesis that it is the insight-related internal reward in NAcc, we tested wether the emotional component (“How strong was your positive emotional response upon solution?”) of the AHA! experience uniquely predicts NAcc activity during insight on a trial by trial basis. For this, we repeated the single trial analysis described further above by replacing the continuous measure of AHA! with a combination of its three components including their interactions (see equations below).

I. Beta value ~ acc + trial# + ROI + run + (1|ID) + (1|item) + Ɛ.
II. Beta value ~ acc + trial# + ROI + run + Emo + (1|ID) + (1|item) + Ɛ.
III. Beta value ~ acc + trial# + ROI + run + Emo + Certain + (1|ID) + (1|item) + Ɛ.
IV. Beta value ~ acc + trial# + ROI + run + Emo + Certain + Sudden (1|ID) + (1|item) + Ɛ.
V. Beta value ~ acc + trial# + ROI + run + Emo × Certain + Sudden (1|ID) + (1|item) + Ɛ.
VI. Beta value ~ acc + trial# + ROI + run + Emo × Certain × Sudden (1|ID) + (1|item) + Ɛ.

*Note*. *acc* accuracy, *trial#* trial number, *Emo* positive Emotion, *ID* subject.

#### Control analyses

**Figure 3:**
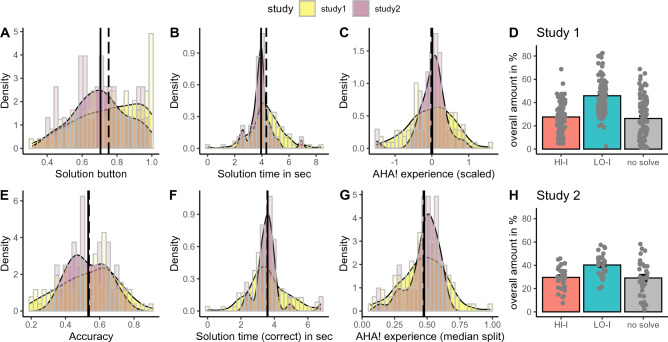
Performance and insight parameter distribution between samples from Study 1 and 2. The dashed line represents the mean average for Study 1 and the full line is the average for Study 2. *HI-I* = trials solved with high accompanied insight; *LO-I* = trials solved with low accompanied insight. Solution time (correct) in sec = Solution time for correctly solved trials. AHA! experience (median split) = Likelihood to solve a trial with accompanied high insight (based on median split) for only correct trials.

We performed a series of additional control analyses to show that the participant samples from Study 1 and 2 do not stem from the different populations and are therefore reasonably comparable (see Fig. 3 and Table 1). Possible differences in main parameters such as likelihood for a solution button press, accuracy, solution time and the AHA! experience between both samples were statistically compared using (general) linear mixed models. The baseline model only included item and subjects as random intercepts and the full model additionally included a binary factor indicating the sample (Study 1 or 2) as predictor. Statistical significance of the study variable was estimated using log-likelihood tests. The results are reported in Table 1.

**Table 1:** Comparison of samples from Study 1 and 2.

	Study 1	Study 2	Chi^2^/p
Solution button	75.2%	70.6%	2.09/0.15
Accuracy	53.7%	53.3%	0.003/0.95
Solution time (s)	4.2	3.9	0.806/0.37
Solution time (correct)	3.4	3.5	0.105/0.75
AHA! [HI-I]	37.5%	42.4%	1.17/0.28
AHA! [HI-I] (correct)	46.2%	48.2%	0.069/0.79
Age (years)	30.2	25.2	**11.69/0.001**
Gender (% female)	64%	63%	0.020/0.89

*Solution button* likelihood to press solution button. *AHA! [HI-I]* median split for AHA! Experience; *(correct)* for correctly solved items.

## Results and discussion

### Study 1—online experiment

Subjects solved 75.2% (*SD* = 18.3%) of all Mooney images and they solved 53.7% (*SD* = 15.9%) correctly in total. On average, solution time was 4.2 s (*SD* = 1.2 s) for all items and 3.4 s (*SD* = 1.1 s) for correctly solved items. Subjects reported to have experienced an *HI-I* in 37.3% (*SD* = 13.7%) of all solved trials and in 47.1% (*SD* = 16.8%) of all correctly solved trials. Consistent with previous research, *HI-I* trials were solved significantly more often correctly compared to *LO-I* (z = 18.34, p < 0.001, odds ratio(*HI-I*) = 3.27, 95% CI [2.88–3.71])^46,47, 60^. For this reason, accuracy is entered as covariate into all further analyses. After the solution, the risk payout option was chosen in 41.4% (*SD* = 33.3%) of all bonus choices and they needed 1.5 s (*SD* = 0.7 s) on average to make this choice.

Importantly, insight significantly predicted subsequent bonus choice (*Chi*^*2*^(1) = 9.41, *p* < 0.005, odds ratio (*HI-I*) = 1.18, 95% CI [1.06,1.32]) when controlling for accuracy and the trial number. Participants were more likely to choose the risk payout (over the fixed payout) when they correctly solved a Mooney image with *HI-I* (38%) compared to solving with *LO-I* (33%) (see Fig. 2A). The results remained significant when predicting bonus choice with the continuous variable of the AHA! experience (*Chi*^*2*^(1) = 13.20, *p* < 0.0002, odds ratio (*HI-I*) = 1.09, 95% CI [1.04, 1.14]). Therefore, the more strongly participants rated to have had an AHA! experience the more likely they were to choose the risk payout (see Fig. 2B). Those results confirm our hypotheses and extend previous research that found the AHA! experience increases risky decision making from a word association task^10^ to a Mooney identification task.

The effect of insight is significant even if we included participants who do not vary their insight ratings or bonus choices. The model for the full 158 participants was reported in Table S1.

### Study 2—fMRI experiment

On average, participants solved 70.6% (*SD* = 15.2%) of all presented Mooney images, and 53.3% (*SD* = 11.1%) of all images were solved correctly. Participants took 3.9 s (*SD* = 0.8 s) to solve all images on average and 3.5 s (*SD* = 0.7 s) for the correctly solved images. The images were solved with high insight (*HI-I*) in 42.4% (*SD* = 9.5%) of all cases and in 48.2% (*SD* = 11.4%) of all correctly solved. Similar to Study 1 and previous research, accuracy was higher (*Chi*^2^(1) = 89.49, *p* < 0.001, odds ratio [*LO-I*] = 0.33, 95% CI [0.26, 0.41]) when the Mooney image was solved with *HI-I* (*M* = 90%) compared to *LO-I* (*M* = 74%)^46,47^. For this reason, accuracy was included as a covariate of no interest into all further fMRI analyses.

#### ROI-analysis

The binary measure of the AHA! experience (*HI-I vs LO-I*) significantly predicted BOLD activity in NAcc during solution (*Chi*^*2*^(1) = 16.78, *p* < 0.0001; *ß* (HI-I) = 0.37, 95% CI [0.24, 0.59]) when controlling for run, accuracy and ROI (left, right). Posthoc analyses revealed that *HI-I* trials (beta = 0.083, 95% [0.03,0.13]) are associated with more BOLD activity in NAcc than *LO-I* trials (beta = − 0.037, 95% [− 0.09, − 0.02]) (see Fig. 2C,D, Table 2). Similarly, the continuous measure of the AHA! experience also positively predicted BOLD activity in the NAcc on a trial by trial basis (*Chi*^*2*^(1) = 97.21, *p* < 0.0001; *ß* = 0.15, 95% CI [0.12, 0.18], see Fig. 2-E, Table 2) when controlling for trial number, accuracy and ROI (left, right). Those results confirm our hypotheses on increased activity in the NAcc during insightful problem solving.

**Table 2:** Influence of AHA! experience on BOLD activity in Nucleus Accumbens (NAcc).

Predictors	Beta estimate (NAcc)					
	Model: binary AHA!			Model: continuous AHA!		
	Beta	CI	p	Beta	CI	p
(Intercept)	− 0.14	− 0.33–0.05	0.663	0.04	− 0.04–0.13	0.907
AHA! [HI-I]	**0.37**	**0.20–0.55**	** < 0.001**			
AHA! (continuous)				**0.15**	**0.12–0.18**	** < 0.001**
accuracy [correct]	0.07	− 0.03–0.17	0.162	0.01	− 0.01–0.04	0.324
run	− 0.11	− 0.19 to − 0.04	0.004			
trial#				0.01	− 0.02–0.03	0.512
ROI [right]	− 0.10	− 0.26–0.05	0.192	− 0.08	− 0.13 to − 0.03	0.002
Random effects						
σ^2^	0.08			0.79		
τ_00 subject_	0.01			0.04		
τ_00 Item_				0.01		
ICC	0.14			0.06		
N_subject_	32			32		
Marg. R^2^/cond. R^2^	0.066/0.20			0.027/0.084		

*Std. Beta* standardized Beta estimates, *CI* 95% CI, *p* p-value, *ICC* intraclass coefficient, *Marg.R*^*2*^*/cond. R*^*2*^ marginal and conditional R^2^, *AHA! [LO-I]* AHA! Experience (for solved trials with low accompanied insight), *trial#* trial number.

When testing the relationship between individual components of the AHA! and NAcc activity, *positive emotion* alone was a significant predictor for NAcc activity during insight (*Chi*^*2*^(1) = 6.22, *p* < 0.013, ß = 0.05, 95% [0.01, 0.08]) over and beyond *suddenness* and *certainty*. The exploratory analysis revealed a model with the best fit (*Chi*^*2*^(1) = 6.05, *p* = 0.014, *ß* = 0.04, 95% CI [0.01, 0.06]) additionally assuming an interaction between *positive emotion* and *certainty* suggesting that NAcc activity is highest, when participants rate their solution to be accompanied by a combination of high *emotion* and high *certainty* that it is correct (see Fig. 2F, Table S2, Supplements). The three-way interaction between *emotion, certainty* and *suddenness* did not reach significance (p > 0.71) but *suddenness* alone also explained unique variance in NAcc activity (ß = 0.05, 95% CI [0.01, 0.08], *p* < 0.01). Those results confirm our hypotheses that NAcc activity is uniquely related to the internal reward (*positive emotion*) component of insight, although all three components (*suddenness, certainty* and *positive emotion*) and an interaction between *emotion* and *certainty* also contribute to the NAcc activity.

For exploratory purposes, we report a whole-brain analysis to investigate the potential activation of other brain regions during *HI-I* > *LO-I *(see the Supplements and Fig. S1). While the primary cluster was found in the ventral striatum, mostly encompassing the NAcc, amygdala, nucleus caudatus and olfactory bulb, additional clusters were observed in the anterior and posterior cingulate cortex, as well as the bilateral angular gyrus resembling patterns seen in the default mode network. For a more in-depth description of the whole-brain analyses and the results, please refer to^36^.

### Control analyses—comparison of study samples

Because Study 1 and 2 were conducted separately, we performed control analyses to test whether the populations in the two studies are comparable. The results are reported in Fig. 3 and Table 1. Both samples showed a similar gender proportion but sample 1 is on average five years older than the fMRI sample from Study 2. Importantly, no significant differences were found in any of the performance metrics. The proportion of produced events (i.e. *HI-I, LO-I* and not solved items) was comparable between both samples (see Fig. 3D,H). The internet sample from Study 1 showed a more skewed distribution towards pressing the solution button than sample from Study 2. However, both samples produced similar distributions for correctly solved trials (accuracy).

In sum, given that the two samples do not significantly differ in any of the performance metrics, we believe the fMRI finding of insight-related NAcc activity in Study 2, generalises to the sample of Study 1, and it is reasonable to assume that NAcc contributes to the insight-related increased risky decision making finding in that Study 1.

## General discussion

People feel more motivated after finding a solution accompanied by insight^61^ and the presence of insight can impact subsequent decision making. In particular, recent evidence suggests that the subjective AHA! experience is associated with subsequent decision making towards riskier (high reward, high uncertainty) choices unrelated to the insight^10^. Here, we investigated the motivational role of AHA! in decision making and investigated its generalizability to a different insight-eliciting task and its neural mechanism. Because risky decision making has been widely associated with dopaminergic signal transmission in the ventral striatum, specifically the Nucleus Accumbens (NAcc^18,62–65^), we selected this region as our region-of-interest. In a preregistered online study, we first replicated the behavioural effect of insight-related increase in risk-taking using a Mooney identification paradigm. Participants were more likely to choose a risk payout over a fixed payout after solving the problem with a high compared to low AHA! experience. In a second study, we tested whether insight is associated with increased dopaminergic activity in NAcc. For this, we used the BOLD signal in NAcc as a proxy for dopaminergic signal transmission^17,37, 39, 66^ and employed data from an fMRI study^50^ that used the same Mooney identification paradigm as in the behavioural Study 1. Consistent with our hypothesis, we found greater BOLD activity in NAcc when subjects solved the Mooney image with high compared to low AHA!. Importantly, NAcc activity was related to the (positive) emotional component of the AHA! experience associated with internal reward, although the feeling of suddenness and certainty also explained individual variance in this brain region. In sum, across both studies, we found preliminary behavioural and neural evidence supporting insight as an internal reward signal in the NAcc that can promote risk-seeking behaviour possibly via dopaminergic signal transmission.

### Insight influences subsequent decision making

The results of Study 1 demonstrate the generalizability of the insight-related risk decision making effect first reported by Yu et al.^33^ to different stimuli and a different paradigm: Whereas Yu et al.^33^ used a compound remote associate task, which primarily depends on verbal processing, Study 1 used the Mooney identification task, which mainly depends on visual processing. A second major difference between the two studies was the bonus choice design. In Yu and colleagues^33^, the risk payoff was 50/50, but in the current study, we presented a skewed risk choice with 20% of high payout and 80% of zero payout. This low-probability high payout emphasised the role of AHA! in promoting reward-seeking at the cost of certainty, i.e. risky decision making. Despite these differences, participants in both studies were more likely to choose the risk payout after an AHA!, demonstrating the robustness of the insight-related risky decision making effect.

Although the insight effect on risky decision making has not been well studied in the past, researchers have demonstrated other behavioural impact of insight. While insight is usually accompanied by correct ideas^45^, it can also bias the perceived truthfulness of an idea or fact, particularly when it arises in close temporal proximity to an AHA! experience making a false fact seem more true^7,8^. Furthermore, insight also increases memory for the solved problem and its content^6,44,67^. The behavioral impact of insight is thought to be related to the emotional component of insight^35,36,44^. Taken together, evidence from various paradigms has converged, consistently pointing towards the significant behavioral impact of insight.

### Insight as an internal reward signal in NAcc

In general, the AHA! experience has been argued to represent an internal reward signal of having found the solution, supported by neuroscientific evidence^9,32, 68^. For this reason, we assumed that the mesolimbic reward system, specifically the NAcc, as consistently linked to motivation and reward processing (for review^11^, should be more strongly increased for solutions with a high compared to low AHA! experience. Consistent with our expectations, we observed increased NAcc activity related to insight and specifically to the positive emotional aspect of the AHA! experience, further substantiating the notion of a reward-driven mechanism.

Furthermore, during our exploratory whole-brain analysis, we identified additional dopaminergic target regions which have been discussed in the context of creativity and insight, including the prefrontal cortex (specifically anterior cingulate) as well as the nucleus caudatus^32,69^. Interestingly, we also observed insight-related increased activation in brain areas belonging to the default mode network (DMN). The DMN is responsible for facilitating spontaneous cognition, has been causally linked to idea generation and creative thinking^70^ and is susceptible to modulation by dopamine^71–73^.

### Dopaminergic NAcc activity influences subsequent decision making

NAcc activity, particularly its dopaminergic signal transmission, is not only associated with reward processing, but also causally linked to subsequent risky decision making. For example, Zalocusky and colleagues^65^ found that neuronal activity in D2 receptor-expressing cells in the NAcc predicted subsequent decisions and optogenetic stimulation of these cells could immediately transform risk-seeking rats into risk-averse rats, suggesting a causal role of those cells in risk preference. Stopper et al.^63^ found that D1 receptor blockade in NAcc decreased preference for larger, uncertain rewards in well-trained rats. The influence of dopamine on choice behaviour has been explained by modulating the attractiveness of risky options^24,27^. There is also evidence from fMRI studies in humans that show a correlation between NAcc activity and risk-taking behaviour. Matthews and colleagues^74^ observed that activation in the NAcc was greater during deliberations preceding the selection of risky responses than during safe responses in healthy participants. Finally, Knutson et al.^17^ investigated how incidental cues associated with rewards can impact one's willingness to take financial risks. Their findings revealed that when individuals anticipated reward cues, they were more likely to engage in financial risk-taking. This effect was partially explained by increased NAcc activation, suggesting a connection between reward anticipation and increased risk-taking behaviour.

### Alternative explanations for insight-related NAcc activity

Because we did not measure dopaminergic activity in NAcc directly, the observed BOLD signal in NAcc may reflect a signal transmission related to other neurotransmitters as not all reward-related processes in NAcc are mediated by dopamine. Even though dopamine release in the NAcc has been linked to neuronal firing^75,76^ and an increase in the BOLD signal is mediated by postsynaptic D1 receptors^38^, this measure could also be related to signal transmission induced by glutamate^77^ or serotonin^78^.

Furthermore, the observed insight-related NAcc activity is likely not solely indicative of internal reward or the subjective value of a stimulus but may also reflect the salience or perceived novelty of the stimulus^79–82^. This is consistent with our findings, that NAcc activity was not exclusively explained by insight-related positive emotions likely reflecting internal reward. *Suddenness* as well as *certainty* about the solution’s correctness and its interaction with *positive emotions* also uniquely contributed to variance in NAcc activity. This suggests that NAcc activity in this context does *not only* reflect internal reward but likely a combination of different cognitive processes related to insight.

## Limitations

One of the limitations of the current study is that we did not directly measure the link between insight-induced risky decision making and BOLD activity in the NAcc in the same experiment. As discussed above, there has been extensive evidence from animal and human studies supporting the causal influence of dopaminergic NAcc activity on risky decision making. Therefore, it is reasonable to assume that the insight-related NAcc activity demonstrated in Study 2 contributes to the insight-related risky decision making bias observed in Study 1. To further support this assumption, control analyses showed no difference in performance metrics between them (see “Results”—Control analyses, Study 2, Table 1, Fig. 3), suggesting that the neural mechanisms of insight did not differ between the Study 1 and 2. However, to move towards a more direct link between the effects of insight on NAcc activity and subsequent risk decisions, future work could adopt an insight paradigm with bonus choice in the scanner, and perform a mediation analysis between insight, NAcc activity and bonus choice on a trial-by-trial basis. To achieve a causal link, one could combine such a paradigm with dopamine agonists and antagonists.

Furthermore, the influence of insight on subsequent risky decision making is rather subtle as indicated by the small effect size (see marg. R^2^ = 0.3–0.5%, see Table 3). This is partially due to the high variability in the risk choices. People’s behavior in risk-eliciting tasks are malleable and noisy^40,83^. To further exacerbate the noisy behavioral measure, our estimation is based on data from an online study, lacking control of the testing environment. The latter may also explain the high proportion of participants that do not switch in their bonus choice, leading to a high number of subjects that had to be excluded (n = 54), similar to previous work^33^. In the future, it will be useful to replicate the behavioural findings in Study 1 with an in-person experiment for more adequate effect size estimates and less drop-outs.

**Table 3:** Influence of AHA! experience on bonus choice (risky decision making).

Predictors	Bonus choice					
	Model: binary AHA!			Model: continuous AHA!		
	OR	CI	p	OR	CI	p
(Intercept)	0.65	0.39–1.07	0.092	0.39	0.24–0.65	< 0.001
AHA! [HI-I]	1.18	1.06–1.32	**0.002**			
AHA! (continuous)				1.09	1.04–1.14	** < 0.001**
Accuracy [correct]	1.16	0.98–1.38	0.085	1.10	0.92–1.32	0.293
Trial#	1.00	0.99–1.00	0.016	0.91	0.85–0.98	0.012

Random effects						
σ^2^	3.29			3.29		
τ_00_	5.56_subject_			5.56_subject_		
ICC	0.63			0.63		
N	103_subject_			103_subject_		
Marg.R^2^/Cond. R^2^	0.003/0.629			0.005/0.630		

*OR* Odds Ratio, *CI* 95% CI, *p* p-value, *ICC* intraclass coefficient, *Marg.R*^*2*^*/Cond. R*^*2*^ = marginal and conditional R^2^, *AHA! [HI-I]* AHA! Experience (for solved trials with low accompanied insight).

## Conclusion

In sum, the study sheds light on the role of internal reward signals after problem solving influencing our choices. Despite its limitations, this study offers initial evidence for a mechanistic explanation of the previously observed risky decision making bias after insight solutions. This explanation is strongly supported by prior research conducted on both animals and humans. It suggests that the AHA! experience represents reward-related activity in NAcc likely related to dopaminergic signal transmission modulating subsequent risky decision making. Further research is necessary to corroborate those results.

### Supplementary Information

Supplementary Information.

## Data Availability

The datasets generated during and/or analysed during Study 1 & 2 including the R code have been made publicly available and can be accessed at https://github.com/MaxiBecker/Insight_Risk_NAcc.
