# Supplementary material for: The influence of insight on risky decision making and nucleus accumbens activation
Source: Sci Rep. 2023 Oct 11;13:17159. doi: 10.1038/s41598-023-44293-2 (PMC10567742; doi:10.1038/s41598-023-44293-2)
Supplement: Supplementary file 1 — Supplementary Information. [file 41598_2023_44293_MOESM1_ESM.pdf]

Supplementary material

Description of the bonus options in study 1:

Additionally, you can make a bonus choice after each puzzle you solve. In the next two screens you will be shown which options are available to you. Please read those explanations carefully!

CAUTION: If you choose the latter option, you will be given the bigger payment with a 20% probability! With an 80% probability, you will get no payment. How high the bigger payment X is depends on the computer randomly picking one value (staying the same throughout the experiment).

Below you can see an example where the two options are shown as payment charts: Here you have the choice to pick the fixed payment (FIXED) and your bonus will be 6 cents; if you choose the 80/20 draw (DRAW), your bonus will be either 0 cents with an 80% probability, or 45 cents with a 20% probability.

“Here you can see 5 different examples where X differs.

Each solved puzzle will be followed by a bonus choice in which you can choose between a fixed payment (FIXED) of 6 cents OR a bigger payment (DRAW) of X cents.

For each of these 5 examples, please choose whether you would prefer the FIXED or the DRAW choice. Most people tend to prefer the DRAW the higher the amount of X becomes.

What about you? Do you prefer a FIXED bonus of 6 cents or a DRAW between two amounts shown below with an 80/20 chance? Click on the payment option that you prefer.”

Each solved puzzle will be followed by a bonus choice where you can choose between a payment of a fixed amount or a random 80/20 draw between two different amounts (0 cents or X cents).

Table S1.

Influence of AHA! experience on bonus choice (entire online sample)

	**Bonus choice**					
	Model: binary AHA!			Model: continuous AHA!		
Predictors	OR	CI	p	OR	CI	p
(Intercept)	0.24	0.11 – 0.52	**<0.001**	0.15	0.07 – 0.31	**<0.001**
AHA! [HI-I]	1.18	1.06 – 1.32	**0.002**			
AHA! (continuous)				1.09	1.04 – 1.15	**<0.001**
accuracy [correct]	1.17	0.98 – 1.39	0.085	1.10	0.92 – 1.32	0.291
trial#	1.00	0.99 – 1.00	**0.016**	0.91	0.85 – 0.98	**0.011**
Random Effects						
σ^2	3.29			3.29		
τ_{00}	18.59			18.53		
ICC	0.85			0.85		
**N** $_{\textbf{subject}}$	156			156		
Marg.R^2/Cond.R^2	0.001 / 0.850			0.002 / 0.850		

Note. OR = Odds Ratio; CI = 95% confidence interval; p = p-value; ICC = intraclass coefficient; Marg.R^2 / Cond.R^2 = marginal and conditional R^2; AHA! [LO-I] = AHA! Experience (for solved trials with low accompanied insight)

Table S2.

Influence of insight components on BOLD activity in Nucleus Accumbens

	Beta estimate (NAcc)		
Predictors	Std. Beta	CI	p
(Intercept)	0.03	-0.06 – 0.11	0.562
Certain	0.11	0.07 – 0.15	**<0.001**
Emotion	0.04	0.01 – 0.08	**0.016**
Suddenness	0.05	0.01 – 0.08	**0.006**
Certainty × Emotion	0.04	0.01 – 0.06	**0.013**
accuracy	0.01	-0.02 – 0.04	0.489
run	-0.03	-0.11 – 0.04	0.369
trial#	0.01	-0.02 – 0.03	0.635
ROI [right]	-0.08	-0.13 – -0.03	0.002
Random Effects			
σ^2	0.79		
$\tau_{00\ ID}$	0.04		
$\tau_{00\ Item}$	0.01		
ICC	0.06		
N_{ID}	32		
Marg.R^2 / Cond.R^2	0.029 / 0.086		

Note. Std. Beta= standardized Beta estimates; CI = 95% confidence interval; p = p-value; ICC = intraclass coefficient; Marg.R^2 / Cond. R^2 = marginal and conditional R^2; trial# = trial number. NAcc = Nucleus Accumbens.

Exploratory whole brain-based analysis

We additionally report a *whole brain analysis* to investigate whether there are other relevant brain areas predicting *HI-I* > *LO-I* (see Becker, Sommer & Cabeza, 2023 for more details).

First Level Analysis. We used the beta values that had already been estimated for the first level analysis (see further above in section Region-of-interest analysis*).* For each individual, simple contrast t-images were calculated from the beta weights of the onset regressors.

Second Level Analysis. We included the contrast images corresponding to both event types (*HI-I, LO-I*) during the solution in a random-effects analysis using SPM's Full Factorial design. To correct for multiple comparisons, a threshold of p-FWE < .05 (height threshold: t=5.11) was applied at the voxel level with an extent threshold: k=7 voxels. All anatomical areas were determined using the AAL3 atlas (Rolls et al., 2020; Tzourio-Mazoyer et al., 2002) based on the percentage of voxels belonging to a respective anatomical area intersected by an activated cluster from the Full Factorial analysis in SPM.

Exploratory whole brain-based results

The contrast *HI-I* > *LO-I* revealed five bigger clusters (see Fig.S1). The main cluster (1045 voxels) was situated around the ventral striatum including bilateral nucleus accumbens, amygdala and olfactory bulb but also including bilateral putamen, right caudate, orbitofrontal cortex and hippocampus. Another cluster was situated in the posterior cingulate cortex including bilateral middle cingulate cortex and precuneus while there was also a more anterior cluster situated in anterior cingulate cortex and medial orbitofrontal cortex. Finally there were two clusters situated around the left and right angular gyrus.

The reverse contrast (*LO-I* > *HI-I*) revealed four main clusters situated in bilateral middle and left inferior occipital cortex as well as right superior parietal cortex.

For more details on whole brain results see Becker, Sommer & Cabeza, 2023.

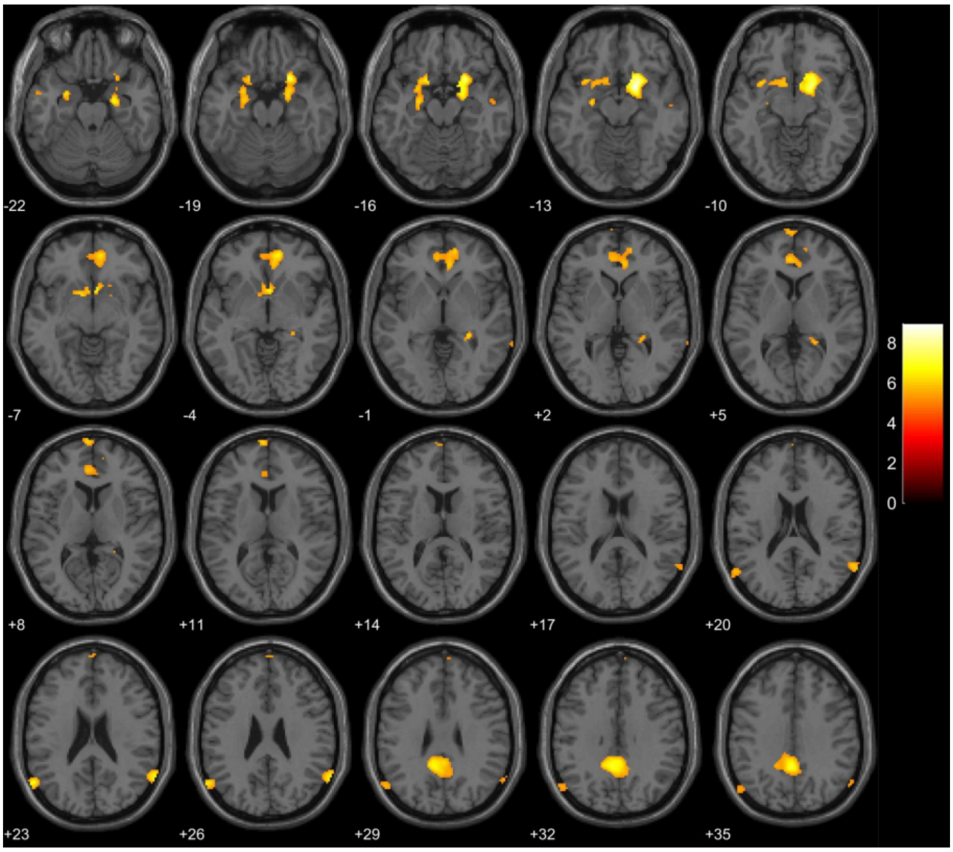

Figure S1. Whole brain activation for contrast High > Low Insight

Note. Threshold for visualisation: voxel-based p-FWE<.05, extent threshold k= 7 voxels.

Supplement References

E. T. Rolls, C.-C. Huang, C.-P. Lin, J. Feng, and M. Joliot, "Automated anatomical labelling atlas 3," *Neuroimage*, vol. 206, p. 116189, 2020.

N. Tzourio-Mazoyer *et al.*, "Automated anatomical labeling of activations in SPM using a macroscopic anatomical parcellation of the MNI MRI single-subject brain," *Neuroimage*, vol. 15, no. 1, pp. 273–289, 2002.

Becker, M., Sommer, T., & Cabeza, R., "Creativity and memory: Cortical representational change along with amygdala activation predict the insight memory effect." *bioRxiv*, 2023-06. https://doi.org/10.1101/2023.06.13.544774
